# Microbiota Modulation of the Gut-Lung Axis in COVID-19

**DOI:** 10.3389/fimmu.2021.635471

**Published:** 2021-02-24

**Authors:** Gislane Lelis Vilela de Oliveira, Camilla Narjara Simão Oliveira, Camila Figueiredo Pinzan, Larissa Vedovato Vilela de Salis, Cristina Ribeiro de Barros Cardoso

**Affiliations:** ^1^Microbiology Program, Institute of Biosciences, Humanities and Exact Sciences, São Paulo State University (UNESP), Sao Jose do Rio Preto, Brazil; ^2^Food Engineering and Technology Department, Institute of Biosciences, Humanities and Exact Sciences, São Paulo State University (UNESP), Sao Jose do Rio Preto, Brazil; ^3^Department of Biochemistry and Immunology, Ribeirão Preto Medical School, University of São Paulo (USP), Ribeirão Preto, Brazil; ^4^Department of Clinical Analysis, Toxicology and Food Sciences, School of Pharmaceutical Sciences of Ribeirão Preto, University of São Paulo (USP), Ribeirão Preto, Brazil

**Keywords:** COVID-19, inflammation, gut-lung axis, microbiota, probiotics

## Abstract

COVID-19 is an infectious disease caused by the Severe Acute Respiratory Syndrome Coronavirus-2 (SARS-CoV-2), and according to the World Health Organization (WHO), to date, SARS-CoV-2 has already infected more than 91.8 million people worldwide with 1,986,871 deaths. This virus affects mainly the respiratory system, but the gastrointestinal tract (GIT) is also a target, meanwhile SARS-CoV-2 was already detected in oesophagus, stomach, duodenum, rectum, and in fecal samples from COVID-19 patients. Prolonged GIT manifestations in COVID-19, mainly the diarrhea, were correlated with decreased richness and diversity of the gut microbiota, immune deregulation and delayed SARS-CoV-2 clearance. So, the bidirectional interactions between the respiratory mucosa and the gut microbiota, known as gut-lung axis, are supposed to be involved in the healthy or pathologic immune responses to SARS-CoV-2. In accordance, the intestinal dysbiosis is associated with increased mortality in other respiratory infections, due to an exacerbated inflammation and decreased regulatory or anti-inflammatory mechanisms in the lungs and in the gut, pointing to this important relationship between both mucosal compartments. Therefore, since the mucous membranes from the respiratory and gastrointestinal tracts are affected, in addition to dysbiosis and inflammation, it is plausible to assume that adjunctive therapies based on the modulation of the gut microbiota and re-establishment of eubiosis conditions could be an important therapeutic approach for constraining the harmful consequences of COVID-19. Then, in this review, we summarized studies showing the persistence of SARS-CoV-2 in the gastrointestinal system and the related digestive COVID-19 manifestations, in addition to the literature demonstrating nasopharyngeal, pulmonary and intestinal dysbiosis in COVID-19 patients. Lastly, we showed the potential beneficial role of probiotic administration in other respiratory infections, and discuss the possible role of probiotics as an adjunctive therapy in SARS-CoV-2 infection.

## Introduction

The human intestinal microbiota consists of more than a trillion microorganisms in a complex and dynamic ecosystem, regulating the immune system and our entire physiology (1). These microbes play very important functions in the body, including nutritional metabolism, development and modulation of immunity, as well as defense against harmful pathogens (2). In the gastrointestinal tract (GIT), the epithelial barrier protects against the invasion of pathogenic microorganisms and helps keeping tolerance to food antigens, while it may also be associated with systemic and pulmonary immune functions. Once damaged, microorganisms translocate into the bloodstream or lungs and can induce septicemia or acute respiratory distress syndrome (3, 4).

**Figure 1:**
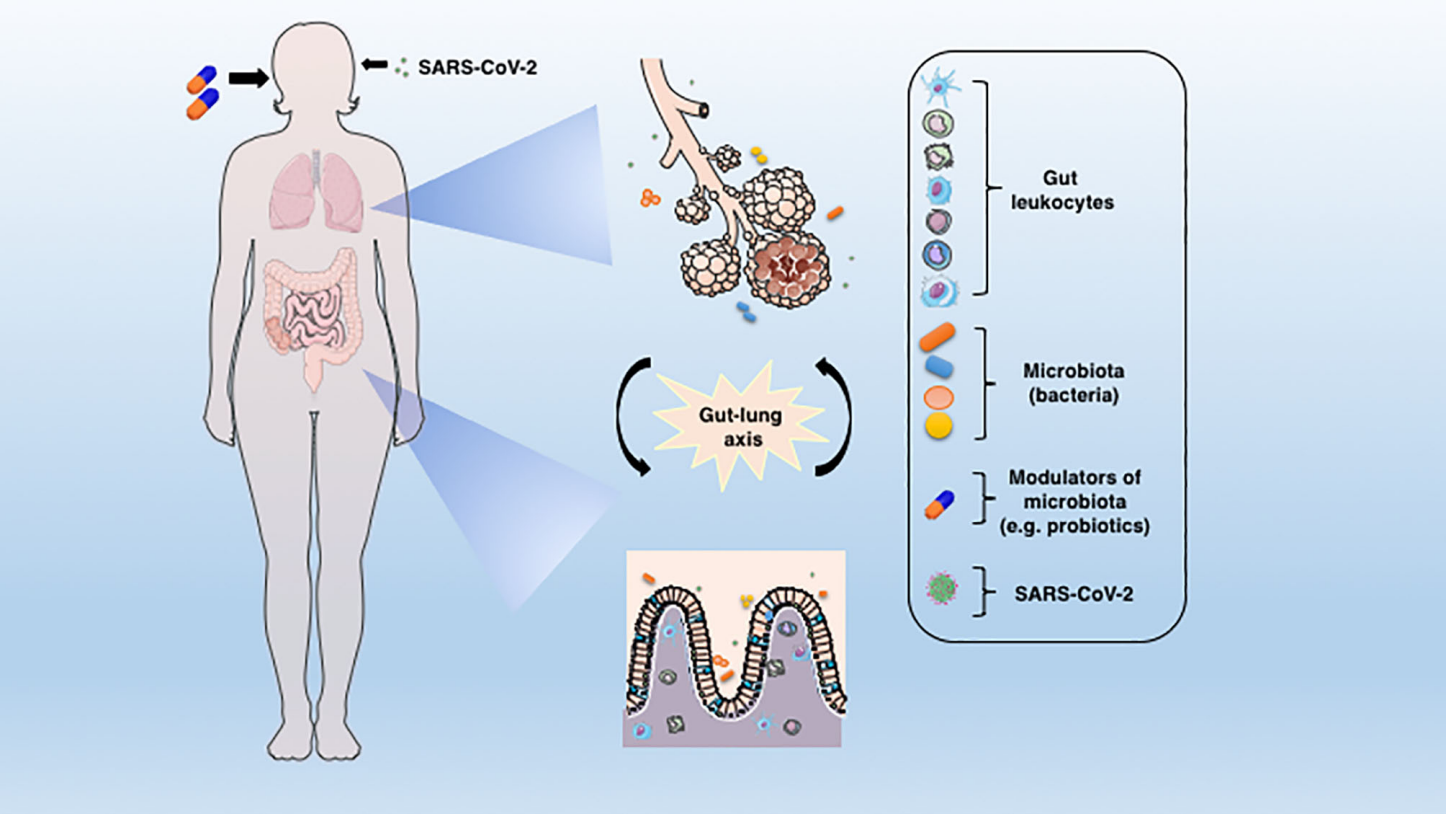
The connection between lung and gut mucosa in the pathogenesis of COVID-19. The SARS-CoV-2 virus infects preferentially cells from the respiratory system, but a large body of evidences points to the GIT as another important target for the virus entry and replication. The dysbiosis, together with the barrier damage and the resulting inflammation may facilitate the disease establishment. The translocated bacteria, leukocytes and the release of inflammatory mediators in the gut-lung axis may contribute to the COVID-19-associated organ deterioration. Some proposed adjunctive therapies such as prebiotics or probiotics, which are aimed at re-establishing the eubiosis state through modulation of microbiota could represent an alternative approach to ameliorate or avoid the worst outcomes of COVID-19.

Indeed, there is evidence for a crosstalk between the respiratory tract and the GIT, or more precisely, between the intestinal microbiota and the lungs (**Figure 1**), and this connection is named the gut-lung axis (5). Changes in the taxonomic composition and decreased diversity and function of the gut microbiota, known as dysbiosis, can affect the immunity of the lungs (6). On the other hand, the respiratory tract has its own microbiota (7) and lung inflammation can lead to intestinal dysbiosis (8). As an example, patients with respiratory infections usually have intestinal dysfunctions (9), which further strengthens the existence of a gut-lung axis. Accordingly, the intestinal microbiota-mucosal immune system interactions and the gut-lung axis have been extensively studied and reviewed in the scientific literature (10–13).

Recent studies hypothesized that endotoxins, microbiota metabolites, cytokines, and hormones from the gut could reach the bloodstream and the lung niche, in a bidirectional gut-lung axis crosstalk (10, 13, 14). Moreover, growing evidence suggests an influence of the gut microbiota metabolites in the migration of bone marrow hematopoietic precursors and inflammation resolution in the lungs (6, 15). In line with that, the host immune status is influenced by intestinal microbiota and may influence the extent of the immunity to viral infections, including the SARS-CoV-2 (16, 17). Due to its essential role in development and maturation of the immune system, in addition to the induction and regulation of immune responses at mucosal surfaces, it is plausible to assume that the microbiota and their metabolites play a significant role in SARS-CoV-2 infection (13, 18, 19).

### Gastrointestinal Tract Arises as a Target to SARS-CoV-2

As already widely known, it is clear that SARS-CoV-2 mainly affects the respiratory system. However, the pathogenicity of the infection is not confined exclusively to the lungs; on the contrary, the virus and the subsequent immune response to it are related to tissue damage on other vital organs while critically ill patients have multiple dysfunction syndromes (20, 21).

Several viruses, such as coronavirus, rotavirus and noroviruses are able to infect the enterocytes from the GIT. The infection impairs the absorption process causing an imbalance in the intestinal function or activation of the enteric nervous system, thus leading to symptoms and important clinical disease manifestations (22–25). Regarding SARS-CoV-2, the GIT is also a target of infection and the virus can be detected in the oesophagus, stomach, duodenum and rectum, besides in the fecal samples of COVID-19 patients (26–28). The main gastrointestinal symptoms commonly seen during COVID-19 are lack of appetite, nausea, vomiting, diarrhea, and abdominal pain (29, 30). In the last months, many researchers have repeatedly shown that the SARS-Cov-2 can, in fact, infect the GIT (**Figure 1**) and there is a high load of replicating viruses, mainly in the gut epithelial cells, as observed in biopsies of the small and large intestine of infected patients (31). Besides, the identification of infectious viruses in fecal samples of COVID-19 patients suggested that the GIT could be a place of viral activity and replication (32, 33). Accordingly, in a work conducted in Singapore, 50% of patients positive to SARS-CoV-2 infection had virus detection in their feces. Still, half of them experienced GIT manifestations such as diarrhea (34). In another study, the presence of SARS-CoV-2 was evaluated in both, throat swabs and fecal samples, through the course of the infection. The feces and respiratory tract swabs were obtained every 1–2 days until two consecutive negative results were reached. The results showed that in that cohort of the patients, fecal samples persisted positive for approximately 5 weeks after respiratory samples tested negative for the virus RNA (35). A similar study reinforced that 80% of a cohort of infected children had positive viral rectal swabs after respiratory tract testing was negative (36). Notably, the live SARS-CoV-2 was also detected in fecal samples from patients who did not have diarrhea, by electron microscopy (37). Moreover, in a preprint study, the analysis of intestinal biopsies showed a long-time persistence of SARS-CoV-2 in the ileum and duodenum of patients after the initial infection (38). Thus, the presence or persistence of the virus in the GIT and stools highlights that SARS-Cov-2 is not limited to the lungs and points to a potential fecal-oral transmission.

Although the specific route through which SARS-CoV-2 infects the GIT is still not fully elucidated, recent reports indicated some possible pathways involved on it. The crucial step of the virus entry into the host organism is through the angiotensin converting enzyme 2 (ACE2) cell receptor (39, 40) and the successful infection also requires the transmembrane protease serine 2 (TMPRSS2), in a cleavage step of the viral S-protein on the host cell membrane, thus allowing efficient viral fusion (41). Both ACE2 and TMPRSS2 have elevated coexpression in the oesophageal upper epithelia and gland cells, besides in the absorptive enterocytes of the ileum and colon from healthy subjects or COVID-19 patients (42, 43). Furthermore, human intestinal epithelial cells (hIECs) can be successfully infected by SARS-CoV-2 and then both intestinal epithelial cell lines and human colon organoids could be potential targets for virus replication, thus potentially contributing to the augmented viremia and spreading of SARS-CoV-2 infection. Importantly, the authors also found that hIECs infected with SARS-CoV-2 are able to promote a strong immune response mainly mediated by type III, but not type I IFNs. The pretreatment of SARS-CoV-2 infected hIECs, with exogenous IFNs, leads to a significant reduction of the infected cells, viral replication, and a sharp decrease in the generation of infectious virus particles. The crucial role of type III IFN in controlling SARS-CoV-2 at the intestinal epithelium was also confirmed by a significant expansion of virus replication after genetic ablation of its specific receptor (22, 44). Hence, although the main manifestations of infection by SARS-CoV-2 are directly linked to the respiratory system, it is necessary to observe GIT alterations that, although less common, also appear during the disease (45, 46).

### Gastrointestinal Manifestations in COVID-19

The presence of gastrointestinal signs or symptoms during COVID-19 is relatively common. In a Chinese province named Zheijiang, it was observed that among 651 patients with a confirmed diagnosis of COVID-19, from January to February 2020, 11.4% had at least one GI symptom, with diarrhea being the most common (8.14%), lasting from 1 to 9 days in most cases, with an average duration of 4 days (28). Though COVID-19 is less frequent in children (47), the percentage of GIT manifestations in this group of patients (13.9%) was very similar to those of adults, according to a study carried out with 244 children in the Chinese city of Wuhan, between January and March 2020 (48).

Patients with gastrointestinal symptoms have a significantly higher rate of chronic liver disease than the patients with COVID-19 but without GIT manifestations (10.81% vs. 2.95%) (28), as well as the transaminases aspartate aminotransferase (AST) (16.5% vs. 5%), and alanine aminotransferase (ALT) enzymes (20.4% vs. 5.9%), that indicate liver damage (30). These subjects are also more likely to have complications of acute respiratory distress syndrome (6.76% vs. 2.08%), progression to the severe and critical forms of COVID-19, more frequently need to use mechanical ventilation and to be admitted in intensive care units (ICU) (6.76% vs. 2.08%) (28), besides prolonged prothrombin time (13.1 vs. 12.5 s) (30). With regard to clinical parameters, patients with gastrointestinal symptoms appear to be more susceptible to fever, fatigue, shortness of breath and headache, which can be caused by increased electrolyte imbalance (28). In comparison with patients with COVID-19 and no gastrointestinal symptoms, those who have these manifestations are also more likely to receive treatment with antibiotics, interferons and immunoglobulins (30). On the other hand, patients with COVID-19 without GIT commitment present a higher incidence of unilateral pneumonia (28) and are twice as likely to recover from the disease compared to those who have digestive symptoms (30.4% vs. 60%) (30).

As COVID-19 becomes more severe, gastrointestinal symptoms become more evident (30). Nevertheless, regarding the markers related to SARS-CoV-2 infection, there is no significant difference in the amount of procalcitonin, C-reactive protein (CRP) (28) and coagulation indicators (except for prothrombin time) between patients with and without COVID-19 manifestation related to the GIT (30). There is also no difference in total blood count or kidney function.

The reasons that explain these differences observed in the course of the disease between patients with and without gastrointestinal symptoms are not completely clear, but it is possible that a viral replication in the tract (30) may lead to a more serious clinical condition. Furthermore, patients presenting extra pulmonary non-classical symptoms of COVID-19 take longer to seek medical help, facilitating the increase in the severity of the disease and making recovery more difficult (30). Apparently, there is no difference in the gender distribution of patients with COVID-19 who have gastrointestinal symptoms (46).

As described before, ACE2 is the gateway to SARS-CoV-2 entry into the host cell (39) and there is high expression of this receptor in the intestine (49), besides in the oral mucosa and in the tongue epithelial cells (50), thus reinforcing the idea that the GIT is also an important target for the virus infection. Indeed, the oral cavity and digestive tract can serve as an infection route for SARS-CoV-2 and the expression of ACE2 in the GIT could explain the presence of gastrointestinal symptoms in patients with COVID-19 (46). Moreover, ACE2 can control intestinal inflammation and diarrhea (51); thus, the interaction between SARS-CoV-2 and ACE2 can lead to a deregulation of this receptor and the intestinal symptoms (49). In addition, since ACE2 was associated with the capture of dietary amino acids, regulation of antimicrobial peptide expression and homeostasis of the intestinal microbiome, it is feasible to assume that ACE2 can be a regulator of the intestinal microbiome and immunity (51). Indeed, as SARS-CoV-2 directly infects the GIT, it is able to generate an inflammatory reaction that can lead to direct and indirect damage on the digestive system (30).

The use of antibiotics is associated with diarrhea (27) and the treatment for COVID-19 may involve the use of these medicines (29), thus generating a change in the composition of the intestinal microbiota (52, 53). This could be another explanation for the diarrhea episodes in patients with COVID-19 and reinforces the hypothesis of the relationship between SARS-CoV-2 and the gut microbiota (54). Thus, it is important to maintain vigilance and pay more attention to the GIT manifestations that appear during COVID-19, as they are less common than the classic respiratory symptoms. Moreover, signals such as diarrhea can not be underestimated because of the risk potential of virus shedding in feces and for early diagnosis of COVID-19 suspected cases (28).

### The Gut-Lung Axis and Dysbiosis in COVID-19

The impact of the intestinal microbiota on systemic immunity and the effect on the respiratory infections has been recently explored in mice and humans (14, 16, 55–59). Studies have demonstrated the essential role of the commensal microbiota in antiviral responses in the lung by modulating the immune responses in homeostatic condition, as well as during the course of the viral infection (16, 55, 60). Notably, researchers have reported a fundamental role of the microbiota on antiviral innate immunity in the respiratory tract, due to its influence on epithelial cells, alveolar macrophages and dendritic cells, also modifying cellular and humoral adaptive immune responses (60, 61).

The intestinal microbiota affects the expression of type I interferon receptors in respiratory epithelial cells, which respond promptly to viral infections *via* IFN-α and IFN-β secretion, restricting the viral replication (60). Macrophages and DCs from germ-free mice failed to produce IFN-α, IFN-β, IL-6, TNF, IL-12, and IL-18 cytokines in response to microbial ligands or viral infection and the natural killer priming becomes defective in the absence of gut microbiota and IFN-I signaling (56). Moreover, using antibiotic-treated mice, it has been showed that comensal microbiota regulate local and systemic IFN-I response through IFN-β secretion by colonic immune cells. More specifically, capsular polysaccharide A derived from *Bacteroides fragilis* induces IFN-β *in vitro* and in colon lamina propria dendritic cells in mice, suggesting that gut microbiota might enhances resistance to viral infections (62). In addition, Ichinohe et al. (16) showed that antibiotic treatment and depletion of gram positive gut bacteria impair the distribution or activation of dendritic cells from the respiratory tract and induce a decrease in migration of DCs from the lung to the draining lymph nodes. Moreover, the intestinal microbiota is also involved in the activation of specific CD4^+^ and CD8^+^ T lymphocytes, in the stable expression of pro-IL-1β, pro-IL-18 and NLRP3, while the inflammasome activation favors the maturation and migration of DCs from the lungs to the draining lymph nodes, after a viral challenge. Abt et al. (55) reported a decreased expression of IFN-γRI, MHC-I, CD40, and CD86 molecules in peritoneal macrophages of antibiotic-treated mice during an early response to viral infection, suggesting that signals derived from gut microbiota modulate the innate immunity prior to viral infection. In experiments with a MERS-CoV animal model, researchers showed the capacity of the virus to induce a decrease on MHC-I and MHC-II expression in macrophages and DCs, impairing the antigenic presentation and leading to defective T cell activation (63).

Some studies have also reported that signals from the commensal microbiota exert different effects on the lung mucosa, such as enhancing the antiviral state in epithelial or innate immune cells and controlling viral replication at the beginning of the infection. The improvement of this innate immunity favors the efficiency of the cellular and humoral adaptive responses in the late course of the infection (16, 55, 60). So, we can assume that beneficial microbes can positively influence the mucosal immune system and promote an efficient response against respiratory viruses (64) (65). The intestinal dysbiosis is associated with the increased mortality in respiratory infections, probably due to a deregulated immune response, with increased secretion of IFN-γ, IL-6, CCL2, and decreased regulatory T cells in the lung and GIT (66).

Four mechanisms have been proposed to explain the impact of the intestinal microbiota on respiratory mucosal immunity: 1) the hypothesis that all mucous tissues are interconnected, that is, the activation of immune cells in a mucosa can influence and reach other distant mucous sites. Thus, the migration of immune cells from the GIT to the mucosa of the respiratory tract may be related to the beneficial impacts exerted by the intestinal microbiota in respiratory viral infections (65, 67); 2) cytokines and growth factors secreted in the GIT mucosa, in response to commensal microbiota, could reach the systemic circulation and act on other mucous tissues (14, 65); 3) the microbial-associated molecular patterns (MAMPs) could be absorbed and conducted to extra intestinal tissues, where they would activate pattern recognition receptors in immune cells and influence innate immune responses (68); 4) the microbiota metabolites absorbed in the gut mucosa have been related to the modulation of mucosal immunity, an effect known as “metabolic reprogramming”. These metabolites, especially short-chain fatty acids, bind to receptors in immune cells of the respiratory tract and enhance the antiviral response in the lung (6, 64).

Regarding COVID-19, it is known that infection of gut epithelial cells by SARS-CoV-2 can induce dysbiosis, intestinal inflammation and gastrointestinal symptoms (31, 69). Furthermore, previous intestinal dysbiosis observed in type 2 diabetes, obesity, hypertension, coronary heart disease, and in other age-related disorders are involved in the deregulation of the inflammatory immune response to SARS-CoV-2, favoring the infection, dissemination and severity in patients with comorbidities (70). The host unbalanced immune response and the massive inflammatory cytokine secretion, known as “cytokine storm” are associated with the disease severity and the worst prognosis in COVID-19 patients (71, 72). In addition, an inflammatory dysbiotic milieu and the epithelial damage induce the expression of ACE2, increasing SARS-CoV-2 replication in the GIT and dissemination to other sites (70). This is consistent with gastrointestinal symptoms and detection of SARS-CoV-2 in anal swabs and fecal samples from COVID-19 patients, even in those subjects negative for viral detection in respiratory swabs or after clearance of respiratory symptoms (31, 73).

As already mentioned, a number of COVID-19 patients had gastrointestinal symptoms (74), a finding that could potentially affect the healthy interactions between the intestinal microbiota and the mucosal immune system, with consequences on the immune response against the lung infection. Moreover, prolonged GIT manifestations, mainly the diarrhea, were inversely correlated to decreased microbiota richness and diversity, associated with immune deregulation and delayed SARS-CoV-2 clearance (75, 76). Studies carried out with COVID-19 patients are demonstrating that, in addition to intestinal dysbiosis, patients may have pharyngeal and pulmonary unbalanced microbiota, reinforcing the hypothesis that mucous surfaces may be connected, and that everything that happens in the GIT mucosa may have consequences on other sites (75, 77–81).

**Figure 2:**
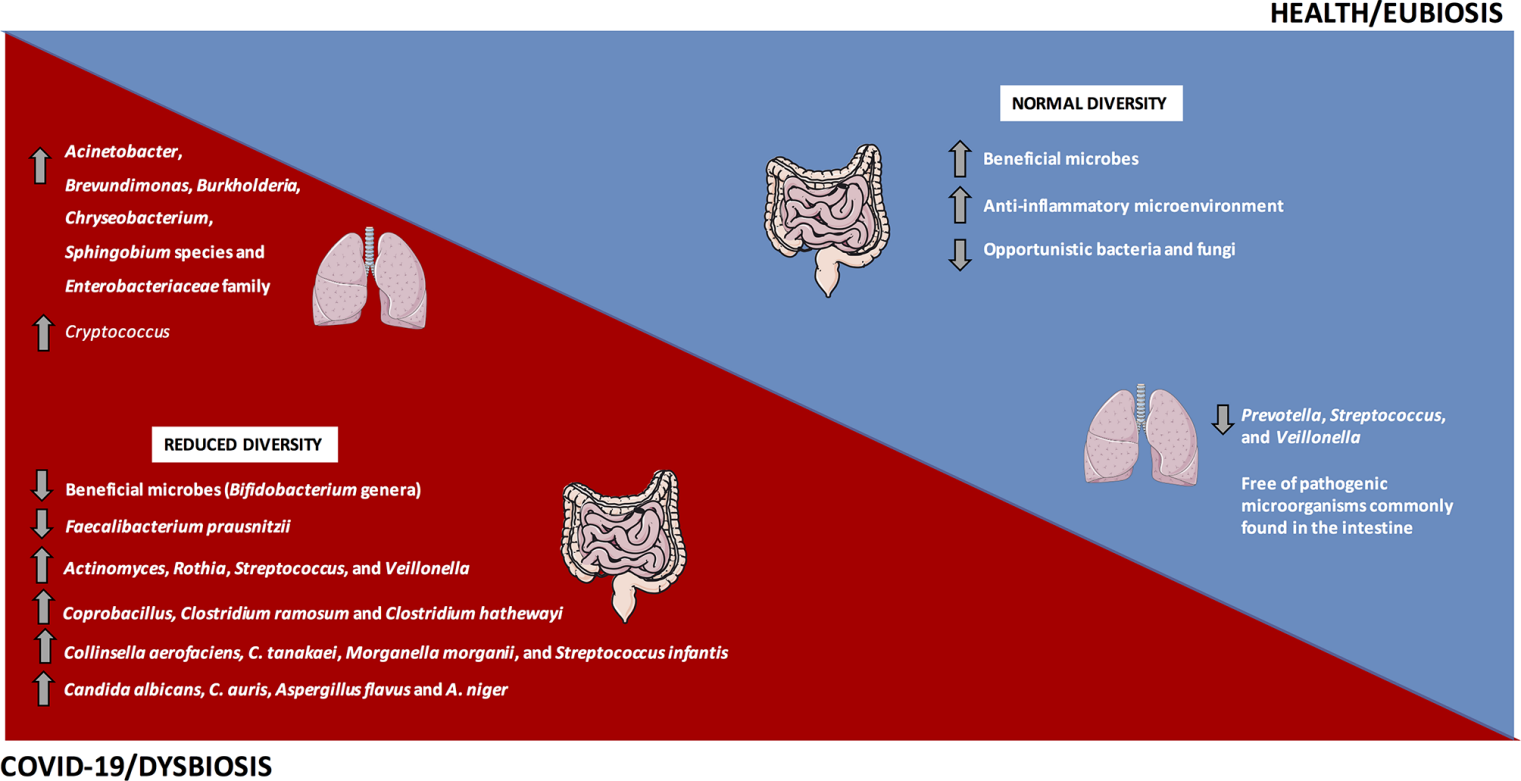
The gut and lung dysbiosis in COVID-19. The frequency and diversity of the gut and lung microbiota are altered in COVID-19 patients, with predominance of the main bacteria and fungi microorganisms depicted in the image. In contrast, a homeostatic environment and the eubiosis state predominate in healthy conditions.

In line with that, in a study carried out in China, Gu et al. (75) evaluated the intestinal microbiota from 30 COVID-19 subjects, 24 H1N1 patients and 30 healthy controls. Subjects infected with SARS-CoV-2 had a decrease in the diversity of the intestinal microbiota when compared to controls, with predominance of opportunistic genera, such as *Actinomyces, Rothia, Streptococcus, and Veillonella*, besides a decrease in the relative abundance of beneficial microbes, such as *Bifidobacterium* genera (**Figure 2**).

Similarly, a pilot study evaluating the intestinal microbiota from 15 hospitalized COVID-19 patients reported significant alterations during hospitalization, with prevalence of opportunistic microorganisms and reduction in beneficial microbes. Even after the disappearance of SARS-CoV-2 and respiratory symptoms’ resolution, the intestinal dysbiosis was still detected. The relative abundance of *Coprobacillus, Clostridium ramosum*, and *Clostridium hathewayi* at baseline positively correlated to COVID-19 severity. In addition, the abundance of *Faecalibacterium prausnitzii*, which favors an anti-inflammatory microenvironment, was inversely correlated to COVID-19 severity (**Figure 2**) (77). During the hospitalization, the relative abundance of *Bacteroides dorei, B. massiliensis, B. ovatus, and B. thetaiotaomicron* that downregulate the ACE2 expression in mouse intestine (82), was inversely correlated to the viral load in feces from COVID-19 patients (77).

In an observational pilot study, Zuo et al. (79) investigated microbiome differences in 15 hospitalized COVID-19 patients and its correlation with the transcriptional profile of SARS-CoV-2. In 46.7% of the subjects, viral RNA was detected in feces, even without GIT manifestations and after clearance of respiratory infection, suggesting a quiescent SARS-CoV-2 infection in the gut and the real possibility of the fecal-oral transmission. Patients with elevated SARS-CoV-2 infectivity demonstrated increased relative abundance of *Collinsella aerofaciens*, *C. tanakaei*, *Morganella morganii*, and *Streptococcus infantis* (**Figure 2**), in addition to the increased carbohydrate metabolism (79). *Morganella morganii* is an opportunistic microbe associated with human infection (83). Patients with decreased SARS-CoV-2 infectivity showed prevalence of *Alistipes onderdonkii, Bacteroides stercoris*, *Lachnospiraceae bacterium*, and *Parabacteroides merdae*, suggesting a beneficial role of commensal microbiota in fighting or competing with SARS-CoV-2 virus in the gut (79).

In another study from the same group, Zuo et al. (78) reported an increased inter-individual fecal mycobiome variation in COVID-19 patients in comparison to healthy controls. Researchers evaluated 30 fecal samples from COVID-19 patients during and after hospitalization and compared to 30 controls by shotgun metagenomics. During all time points of hospitalization, patients with SARS-CoV-2 infection showed increased opportunistic fungi, including *Candida albicans*, *C. auris*, *Aspergillus flavus and A. niger* (**Figure 2**). These last two respiratory pathogens were detected in fecal samples even after SARS-CoV-2 clearance and respiratory symptoms’ resolution, suggesting an unstable intestinal mycobiome and persistent dysbiosis in some COVID-19 patients (78).

Concerning the nasopharyngeal microbiota, De Maio *et al*. (84) analyzed samples from 18 mild COVID-19 patients compared to 22 uninfected controls. The nasopharyngeal microbiota of patients infected with SARS-CoV-2 and controls was similar, i.e., there were no statistically significant differences in the richness and diversity of the samples collected from both groups, suggesting a resilient microbiota in early mild COVID-19. The main phyla detected in samples were Firmicutes, Bacteroidetes, Proteobacteria, Actinobacteria, and Fusobacteria.

In a pre-print work, Budding et al. (80) investigated pharyngeal microbiota from 46 COVID-19 patients, positive for SARS-CoV-2 detection by PCR, and 89 negative ones, and showed that there are two different microbiota clustering, a homogenous microbiota cluster with 75% of the negative samples, and another one, more heterogeneous with 47% of the positive SARS-CoV-2 samples. Older patients exhibited decreased microbial diversity and heterogeneous microbiota, suggesting an age-dependency in pharyngeal dysbiosis and susceptibility to SARS-CoV-2 infection (80). Furthermore, the pharyngeal microbiota might influence the progression of respiratory viral infections through multiple mechanisms, including direct inhibition of viral adherence and mucosal immune response’s modulation (85–88). The status of the pharyngeal microbiota, including the richness and diversity, may affect the SARS-CoV-2 infection susceptibility, the disease progression, and the probability of secondary infections by pathogenic bacteria (80, 89).

The healthy human lungs present decreased density of microbes, including species of *Prevotella, Streptococcus*, and *Veillonella* (90–92). In an observational study, Fan et al. (81) evaluated the lung microbiota in biopsies from 20 fatal cases of COVID-19. *Acinetobacter, Brevundimonas, Burkholderia, Chryseobacterium, Sphingobium* species and Enterobacteriaceae members dominated the lung microbiota in these patients (**Figure 2**). The Enterobacteriaceae family, which comprises species commonly found in the intestinal microbiota and includes some pathogenic microbes, such as *Enterobacter, Escherichia coli, Klebsiella*, and *Proteus*, was detected in the lungs of deceased COVID-19 patients (81). Within the *Acinetobacter* genus, the *A. baumannii* is related to multi-resistant infections and mortality (93). The lung fungal microbiota in COVID-19 patients was dominated by *Cryptococcus* (**Figure 2**), followed by *Aspergillus, Alternaria, Dipodascus, Mortierella, Naganishia, Diutina, Candida, Cladosporium, Issatchenkia*, and *Wallemia* (81). *Cryptococcus* infections were related to high mortality rates in immunocompromised individuals (94), and the *Issatchenkia, Cladosporium*, and *Candida* represent opportunist species involved in mycosis in immunosuppressed patients (81).

Given the crucial role of the intestinal microbiota in the regulation of the immune responses at mucosal surfaces and the maintenance of the systemic and pulmonary health, we believe that microbiota studies are further necessary to improve our knowledge concerning these interactions in context of SARS-CoV-2 infection. The identification of the mucosal microbial communities could help to find biomarkers involved in COVID-19-related dysbiosis, as well as in the determination of potential therapeutic targets for the development of immunobiotics for the treatment of these patients. Indeed, some alternatives for the prevention, diagnosis, prophylaxis and treatment of COVID-19 were already proposed, such as the use of ACE2 receptor inhibitors (95) and the modulation of the intestinal microbiota through the use of probiotics, prebiotics, synbiotics, and postbiotics, alone or in combination, for maintenance of the intestinal ecological balance, prevent secondary bacterial infections and also protect the respiratory system (47, 96). These therapeutic interventions could also improve the immune response in patients affected by comorbidities, and possibly ameliorate the immunity against the SARS-CoV-2 after future vaccinations (97).

### Experimental Therapies Based on Microbiota Modulation

There are a growing number of studies evaluating the effect of probiotic/prebiotics administration in reducing the incidence, duration and severity of viral respiratory infections in humans. The potential for probiotic use is supported by experimental studies, meta-analyses and clinical trials on influenza virus, rhinovirus, and respiratory syncytial virus (98–102). Although the mechanisms have not been determined in SARS-CoV-2 infection, some probiotic strains present antiviral properties in other coronaviruses (103–106).

According to the International Scientific Association for Probiotics and Prebiotics (2013), probiotics is defined as “live microorganisms that, when administered in adequate amounts, confer a health benefit on the host”. Probiotics can be found in fermented foods and in several supplements, but only well-defined strains, with scientifically demonstrated benefit can be used (107). The termed “prebiotic” was coined in 1995 by Gibson and Roberfroid, and the current definition (2016) is “a substrate that is selectively utilized by host microorganisms conferring a health benefit”, i.e., the prebiotic dietary fiber needs to function as substrate for health-promoting microbes in the intestine (108). Moreover, synbiotics are defined as a “mixtures of probiotics and prebiotics that beneficially affect the host” (109). Posbiotics include functional bioactive substances resulting from the microbial fermentation processes, including metabolities such as short chain fatty acids and bacterial cell components, which confer beneficial impact on the host health (110, 111).

Probiotics may have two different immunomodulatory impacts on the host and can induce a pro- or anti-inflammatory immune responses (112, 113). In an immunostimulatory response, there is an increase in the phagocytic activity of macrophages, dendritic cells, and neutrophils, in addition to increased NK cell activity, inflammatory cytokines release and Th1/Th17 polarization in the gut mucosa (114–117). In an anti-inflammatory response, some probiotic strains can induce regulatory T cells, *via* dendritic cell modulation in the gut mucosa, inducing IL-10, TGF-β, and enhancing the IgA secretion and gut barrier function (118–120). Therefore, knowledge of the probiotic strain and experimental studies are essential to determine the best strain to achieve the therapeutic objectives. So, once probiotics can modify the dynamic equilibrium between inflammatory and regulatory mechanisms and impact the viral clearance, the immune response and lung damage, their use might be crucial to dampen the acute respiratory distress syndrome, and prevent major complications of SARS-CoV-2 infection (102, 121).

In experimental murine models, some probiotic *Lactobacillus* strains stimulate the IFN-γ, IL-6, IL-4 and IL-10 secretion in the lungs, and a decrease in *S. pneumonia* and its dissemination to the bloodstream (122). Additionally, *Lactobacillus casei* increases the phagocytic and killing processes in alveolar macrophages, IFN-γ and TNF-α expression, thus helping fight against the influenza virus (123). In humans, a randomized clinical trial using *Lactobacillus plantarum* DR7 reported suppression of plasma concentrations of inflammatory cytokines, such as IFN-γ and TNF-α, and increased IL-4 and IL-10 in young adults with upper respiratory infections (124). Given the cytokine storm observed in COVID-19, this therapeutic approach could benefit the patients by mechanisms such as the reestablishment of gut barrier *via* increased expression of tight junctions and augmented short-chain fatty acids production, including butyrate, which have anti-inflammatory effect and could, theoretically, reduce the SARS-CoV-2 invasion of colonocytes (102).

There are several studies showing the impact of probiotic supplementation in the prevention of upper and lower respiratory tract infections in humans. In a meta-analysis including 12 randomized clinical trials and 3,720 individuals, the probiotic administration reduced the number and duration of acute upper respiratory episodes, the antibiotic duration and disease severity (125). Probiotics have also been used to prevent bacterial lower respiratory infections in critically ill patients. Two meta-analysis including almost 2,000 patients showed that probiotic supplementation decreased the incidence of ventilator-associated pneumonia (126, 127).

Immune senescence and decreased diversity of the intestinal microbiota potentially increased the incidence of infections in the elderly, who are at increasing risk for COVID-19 (128, 129). So, daily intake of fermented foods, containing probiotics could improve the performance of the immune system *via* interaction with the microbiota of the GIT mucosa. In a double-blinded, controlled clinical trial, Guillemard et al. (130) evaluated the effect of dairy product containing *Lactobacillus casei* in 1,072 individuals, with median age of 76 years, during 3 months, and showed that probiotics significantly decreased the incidence and the episodes of upper respiratory infections.

In addition to prevent lower and upper respiratory infections, probiotics could assist in the treatment of diarrhea associated with SARS-CoV-2 infection itself or caused by the antibiotics used to treat secondary pulmonary infections (131, 132). One of the risk factors associated with SARS-CoV-2 infection is secondary bacterial pneumonia. In recent works on COVID-19, the secondary infections were significantly correlated to worst prognosis, outcomes and death (81). A meta-analysis performed by Szajewska et al. (133) by using 18 randomized controlled clinical trials, with 4,208 participants, demonstrated that orally *Lactobacillus rhamnosus* GG probiotic administration was associated with decreased diarrhea duration and reduced hospitalizations in inpatients. Antibiotics induce significant alterations in the intestinal microbiota balance, which may result in antibiotic-associated diarrhea. Probiotics could prevent this condition *via* epithelial barrier reinforcement and restoring eubiosis. Indeed, a meta-analysis including 33 randomized, controlled clinical trials, with 6,352 subjects, demonstrated that probiotic supplementation confer a moderate protective impact on antibiotic-associated diarrhea, reducing its duration (134).

The improvement of the intestinal microecology and the eubiosis process by taking probiotics may promote a regulated immune system and prevent an excessive inflammation or secondary infections (97, 135–137). In accordance, some strains of *Bifidobacterium, Lactobacillus paracasei*, and *L. rhamnosus* reduce the occurrence of respiratory infections, such as H1N1, H3N2, and H5N1 by boosting the vaccine immune responses (67, 138, 139). This improvement of the microbiota-mucosal immune system interactions could also benefit the immune responses to vaccination against SARS-CoV-2 virus. Nevertheless, though we hypothesize that dysbiosis or microbiota modulation could potentially affect the efficacy of COVID-19 vaccines, to date, there are no current published studies regarding the relationship between the gut-lung microbiota and vaccination to this infection. In spite of that, researchers around the world have been continuously working on the search for vaccines against COVID-19 and some of them were recently approved for human use (140–142).

In view of the current knowledge, the modulation of microbiota is being investigated as a possible adjunctive therapy for COVID-19. D’Ettorre et al. (143) evaluated the impact of probiotics to reduce disease progression, in 28 patients. The included subjects presented fever, lung involvement and requested non-invasive oxygen therapy. Patients received antibiotics, tocilizumab, and hydroxychloroquine, alone or combined, and the multistrain probiotic administration (2,400 billion bacteria/day). The probiotic formulation contained *Lactobacillus acidophilus*, *L. helveticus*, *L. paracasei*, *L. plantarum*, *L. brevis*, *Bifidobacterium lactis*, and *Streptococcus thermophilus*. After 3 days of supplementation, all patients in the probiotic group presented remission of diarrhea and other symptoms resolution, when compared to 42 healthy controls. After 7 days, the probiotic group showed a significant decrease in the estimated risk of respiratory failure, and in hospitalizations in intensive care units and mortality, pointing to the important role of the gut-lung axis in the control of the SARS-CoV-2 infection (143). In addition, at the time we write this review, there are approximately 10 clinical trials registered on ClinicalTrials.gov and currently in progress to assess the impact of the use of probiotics and modulators of the gut microbiota on COVID-19. **Table 1** summarizes the main aspects of these clinical trials in COVID-19 patients.

**Table 1:** Ongoing clinical trials testing the effectiveness of gut microbiota modulation in patients with COVID-19 (ClinicalTrials.gov).

Registration	Country	Study design	Evaluation	Target subjects	Intervention	Administration route	Duration	Participants (number)
NCT04458519	Canada	Randomized single-blind, prospective clinical trial	Influence on severity of symptoms	Mild–moderate non-hospitalized COVID-19 patients (18–59 years)	*Lactococcus lactis* W136, (2.4 x 10^9^ CFU)	Intranasal (twice-daily)	14 days	40
NCT04390477	Spain	Randomized open-label, prospective case-control clinical trial	Influence on symptoms, hospitalization duration, and virus clearance	Moderate–severe hospitalized COVID-19 patients (≥ 18 years)	Probiotic strain not informed, (1 x 10^9^ CFU)	Orally (daily capsule)	30 days	40
NCT04366180	Spain	Randomized quadruple-blinded, multicentric clinical trial	Influence on the incidence and severity of COVID-19	Health workers exposed to the virus (≥ 20 years)	*Lactobacillus* K8 strain (3 x 10^9^ CFU)	Orally (daily capsule)	2 months	314
NCT04517422	Mexico	Randomized quadruple-blinded, controlled clinical trial	Influence on disease progression, GIT symptoms, microbiota, viral load, IgG/IgM	Mild-symptomatic COVID-19 patients, SpO2 > 90% (18–60 years)	*L. plantarum* CECT7481, CECT 7484, CECT 7485, *Pediococcus acidilactici* CECT 7483	Orally (daily capsule)	30 days	300
NCT04366089	Italy	Randomized single-blind, prospective clinical trial	Influence on disease progression, hospitalization in intensive care units	Hospitalized COVID-19 patients (≥ 18 years)	Oxygen ozone therapy plus *B. lactis* DSM 32246, 32247, *L. acidophilus* DSM 32241, *L. helveticus* DSM 32242, *L. paracasei* DSM 32243, *L. plantarum* DSM 32244, *L. brevis* DSM 27961 (2 x 10^9^ CFU)	Orally (six sachets/ twice-daily)	7 days	152
NCT04462627	Belgium	Non-randomized, open label clinical trial	Influence on COVID-19 transmission to health care professionals	COVID-19 positive patients and healthy volunteers (≥ 18 years)	*L. acidophilus* NCFM *B. lactis* Bi-07 (12.5 x 10^9^ CFU)	Orally	4 days	500
NCT04399252	United States of America	Randomized double-blind placebo-controlled clinical trial	Influence on the gut microbiome	Exposed household contacts of COVID-19 patients (≥ 1 year)	*Lactobacillus rhamnosus* GG	Orally (two capsules/day)	28 days	1,000
NCT04507867	Mexico	Randomized single-blind controlled- clinical trial	Influence on disease progression, reduction of comorbidities’ complications	Hospitalized COVID-19 patients	Nutritional support system (NSS) plus *Saccharomyces bourllardii* (250 mg)	Orally (two capsules/day)	6 days	240
NCT04403646	Argentina	Randomized triple-blind controlled- clinical trial	Influence on gut microbiota modulation	Hospitalized COVID-19 positive patients (≥ 18 years)	Dry extract polyphenols (tannins)240 mg B12 vitamin 0.72 µg	Orally (two capsules/day)	14 days	140
NCT04540406	United States of America	Randomized open-label, controlled clinical trial	Influence on gut microbiota, early treatment of suspected or confirmed COVID-19 in prediabetes or T2D patients	Mild-moderate COVID-19 patients, prediabetes or T2D patients (18–69 years)	NBT-NM108, a novel botanical-based fixed-combination drug (30 g)	Orally (4 times-daily)	28 days	100

COVID-19, Coronavirus disease; CFU, colony forming unit; GIT, gastrointestinal tract; T2D, type 2 diabetes; SpO2, oxygen saturation.

A prospective case-control, open-label pilot study conducted in Hospital Universitario del Vinalopó, Spain, aims to evaluate the effect of daily oral administration of a probiotic mixture, during 30 days, in symptoms’ improvement, days of hospitalization, and virus clearance in 40 COVID-19 patients (NCT04390477). A randomized single-blinded clinical trial in Canada, at Centre hospitalier de l’Université de Montréal, is planning to examine the impact of twice-daily intranasal administration with *Lactococcus lactis* W136, during 14 days, in 40 non-hospitalized COVID-19 patients (NCT04458519). Moreover, a multicenter randomized, quadruple-blinded clinical trial will investigate the preventive effect of oral administration of *Lactobacillus Coryniformis* K8 (3 x 10^9^ CFU/day) on 314 health professionals exposed to COVID-19 during 2 months (NCT04366180).

At the Hospital General Dr. Manuel Gea Gonzalez, Mexico, a randomized controlled clinical trial will evaluate the safety and efficacy of oral daily supplementation with some strains of *Lactobacillus plantarum* and *Pediococcus acidilactici* CECT 7483, during 30 days, in 300 mild COVID-19 patients. The aims of this study are to evaluate the risk of progression to moderate/severe disease or death, in addition to investigate the frequency and severity of gastrointestinal symptoms, pulmonary involvement, viral load, and the modulation of the fecal microbiota in correlation to clinical improvements (NCT04517422). In Austria, at Medical University of Graz, a randomized quadruple-blinded, placebo-controlled study will investigate the effects of synbiotic oral treatment with *Bifidobacterium bifidum*, *B. lactis*, *Enterococcus faecium*, *Lactobacillus acidophilus*, *L. paracasei*, *L. plantarum*, *L. rhamnosus*, *L. salivarius*, inulin, and fructooligosaccharide (FOS) in 108 volunteers during 30 days. Researchers hypothesized that synbiotic therapy could decrease the diarrhea duration, improve the stool consistency, intestinal inflammation, dysbiosis, and the gastrointestinal symptoms of COVID-19 (NCT04420676). In the United States, a randomized, triple-blinded, placebo-controlled study is being conducted at Duke University to evaluate the effect of oral daily intake of capsules containing *Lactobacillus rhamnosus* GG on the gut microbiota in 1,000 exposed household contacts of COVID-19 patients, during 28 days (NCT04399252).

The role of probiotics and other strategies aiming to modulate the intestinal microbiota in COVID-19 need further investigations, especially randomized, double-blinded, controlled clinical trials, including larger cohorts in different ages and disease courses (64). Not all probiotics should be used to constrain respiratory infections, and the studies’ variations include differences in specific strains, supplementation duration, administration forms, doses and follow-up times (64, 102). Finally, since COVID-19 is essentially an inflammatory disease, the shaping of lung-gut microbiota by the use of probiotics (**Figure 1**) could represent an important adjunctive tool to the control of the excessive inflammation that usually culminate in the worst disease prognosis.

## Conclusions

The mucosal surfaces such as in the lungs and gut play an essential role in the modulation of the immune responses, by combating pathogenic microorganisms and avoiding excessive inflammation or tissue damage. This fine tuning of the local immunity is also dependent on the equilibrium of the local microbiota, while a breakdown in the mucosal tolerance together with a dysbiotic condition may favour the establishment and progression of infections, such as that caused by SARS-CoV-2 virus. Furthermore, since in COVID-19 both respiratory and gastrointestinal mucosas are affected, together with relevant alterations in local microbiota and inflammation, it is plausible to assume that adjunctive therapies based on the modulation of the gut-lung axis and re-establishment of eubiosis could be an important therapeutic approach for constraining the harmful consequences of COVID-19. However, further studies are still necessary to unravel the efficacy of these microbial-based interventions, especially in severe cases of COVID-19.

## Author Contributions

GO designed the study and wrote and reviewed the manuscript. CO, CP, and LS wrote the manuscript. CC designed, wrote, and reviewed the study. All authors contributed to the article and approved the submitted version.

## Funding

This work was supported by CAPES (Financial Code 001), CNPq (310174/2016-3 and 309583/2019-5), and Fundação de Amparo à Pesquisa do Estado de São Paulo (FAPESP 2017/08651.1 and 2021/00229-4).

## Conflict of Interest

The authors declare that the research was conducted in the absence of any commercial or financial relationships that could be construed as a potential conflict of interest.
